# No evidence for *Wolbachia* as a nutritional co-obligate endosymbiont in the aphid *Pentalonia nigronervosa*

**DOI:** 10.1186/s40168-020-00865-2

**Published:** 2020-05-22

**Authors:** Alejandro Manzano-Marín

**Affiliations:** UMR 1062 Centre de Biologie pour la Gestion des Populations, INRA, CIRAD, IRD, Montpellier SupAgro, Univ. Montpellier, 755 avenue du campus Agropolis, Montpellier, France

**Keywords:** Aphid, *Buchnera*, *Wolbachia*, *Pentalonia nigronervosa*, Co-obligate, Symbiont

## Abstract

Obligate symbiotic associations are present in a wide variety of animals with a nutrient-restricted diet. Aphids (hemiptera: Aphididae) almost-universally host *Buchnera aphidicola* bacteria in specialised organs (called bacteriomes). These bacteria supply the aphid with essential nutrients lacking from their diet (i.e. essential amino acids and some B vitamins). Some aphid lineages, such as species from the Lacninae subfamily, have evolved co-obligate associations with secondary endosymbionts, deriving from a loss of biotin- and riboflavin-biosynthetic genes. In this study, I re-analyse previously published sequencing data from the banana aphid *Pentalonia nigronervosa*. I show that the metabolic inference results from De Clerck et al. (Microbiome 3:63, 2015) are incorrect and possibly arise from the use of inadequate methods. Additionally, I discuss how the seemingly biased interpretation of their antibiotic treatment analyses together with an incorrect genome-based metabolic inference resulted in the erroneous suggestion “that a co-obligatory symbiosis between *B. aphidicola* and *Wolbachia* occurs in the banana aphid”.

## Main text

In a previous study, De Clerck et al. [1] claimed to present evidence for a potential co-obligate association of *Buchnera* and *Wolbachia* in the aphid *Pentalonia nigronervosa*. They reach these conclusions mainly based on 4 lines of evidence: (1) the apparently fixed nature of *Wolbachia* in *P. nigronervosa*; (2) a genome-based metabolic inference coming from a pooled metagenomic assembly of extracted DNA from three *P. nigronervosa* populations sampled in Gabon, Madagascar, and Burundi; (3) an antibiotic treatment directed towards the elimination of the endosymbionts; and (4) the attempted detection of putatively missing genes through PCR. In this work, I have re-analysed the publicly available sequencing data and performed a genome-based metabolic inference of the biosynthetic capabilities of *Buchnera*. I find that this *Buchnera* has equivalent biosynthetic capabilities, concerning essential amino acids, B vitamins, and co-factors, to all published *Buchnera* strains from “mono-symbiotic” aphids (only harbouring *Buchnera* as the nutritional obligate symbiont), and thus should not need an additional partner to fulfil its nutritional role. I also critically discuss the interpretation of the experimental results presented by De Clerck et al. [1] and conclude that their suggestion of the nutritional-based co-obligate nature of *Wolbachia* in *P. nigronervosa* derives from inadequate analyses of their data as well as a seemingly biassed interpretation of their experimental results.

**Fig. 1:**
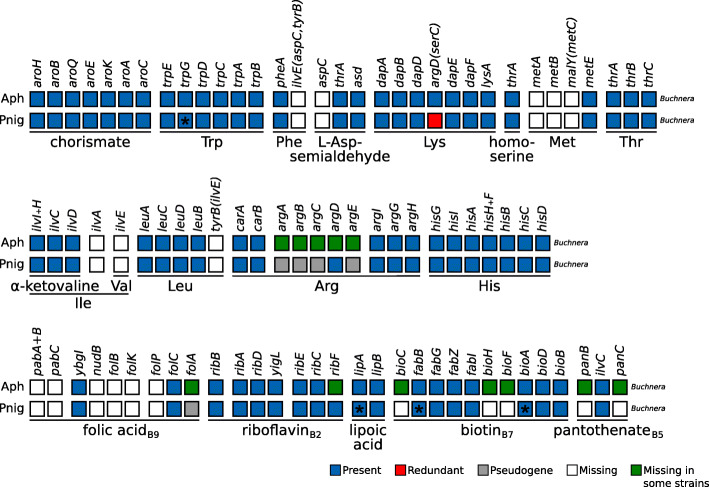
Essential amino acid and selected B vitamin and co-factor biosynthetic metabolic capabilities of obligate symbiotic consortia of different aphid species. Diagram summarising the metabolic capabilities of *Buchnera* from “mono-symbiotic” Aphidinae aphids (Aph) and *P. nigronervosa* (Pnig). “Aph” rows are a collapsed representation of several Aphidinae species (see Supplementary Table S3 in Additional file 1). The names of genes coding for enzymes involved in the biosynthetic pathway are used as column names. Each row’s boxes represent the genes coded by the *Buchnera* genome. On the bottom, lines underlining the genes involved in the pathway leading to the compound specified by the name underneath the line. For amino acids, its three-letter abbreviation is used

To produce a genome assembly, I downloaded the three datasets deposited in NCBI with project number PRJNA268300 and accession SRX766492. The pooled genome assembly of these reads resulted in 269,717 contigs that were then binned into 4 groups: *Wolbachia*, *Buchnera*, mitochondrion, and the aphid host. These resulted in 135 scaffolds for *Buchnera* with an average *k*-mer (77 bp) coverage of 110 and 1309 for *Wolbachia* with an average *k*-mer coverage of 396. The search for the genes involved in the biosynthesis of essential amino acids (EAAs), B vitamins, and other co-factors (hereafter referred to collectively as “nutritional genes”) revealed that *Buchnera* from *P. nigronervosa* retains all genes common to other *Buchnera* from aphids displaying a mono-symbiotic relationship with *Buchnera* (Fig. 1). Additionally, I found that all genes claimed by De Clerck et al. [1] to be missing from *Buchnera* in the biosynthetic pathways shown in Fig. 4 of the article are actually present, except for that of *pgm* (coding for a phosphoglucomutase which is absent in all currently sequenced *Buchnera* strains). The *lipA*, *fabB*, and *bioA* genes show frameshifts in low complexity regions (see GenBank file for details), which is not uncommon for *Buchnera* nor for other small A+T-biassed genomes. The expression of these genes is likely to be rescued by ribosomal frameshifting. Additionally, the *trpG* gene also displays a frameshift in a low complexity region and a stop codon in the consensus sequence. Closer inspection revealed that the “TAG” stop codon shows a variant (“CAG”) present at 13.40% in library SRR1662246. This could be explained by the collapsed assembly of the tandem *trpEG* units found in other Aphidinae, which tend to show pseudogenised variants [2, 3]. To test for the presence of the genes claimed as missing by De Clerck et al. [1] and “nutritional genes”, and in a similar fashion to De Clerck et al. [1], I performed read mapping of each library *vs.* the nucleotide sequence of each gene. This confirmed the presence of most of these genes in all three sequencing libraries (Supplementary Table S1 in Additional file 1, Supplementary Material online). Many *Buchnera* genes had very low coverages of ≤3 in sequencing library SRR1661114 and ≤15 in sequencing library SRR1662249. In fact, these two libraries had between 1 and 2% of the reads coming from *Buchnera*, contrasting library SRR1662249, where 25% of the reads mapped to the *Buchnera* scaffold bin (supplementary table S2 in Additional bluefile 1). The fact that the authors solely searched for intact protein-coding genes, using myRAST [4, 5], and their binning method based on a BLASTX search *vs.* the nr database of NCBI, rather than a narrower database consisting of expected associates, surely impacted both accurate binning and gene identification. Therefore, the genome-based metabolic inference results do not in fact support the nutritional need of a co-obligate symbiont in *P. nigronervosa*. The lack of amplification by PCR of these genes by De Clerck et al. [1] can be explained by the nucleotide sequence divergence between *Buchnera* harboured by not-so-distantly related aphids. Due to this divergence across *Buchnera* strains, using positive amplification of the target gene in a sample from *Acyrthosiphon pisum* was not an adequate control. Manual inspection of the alignments of the primers *vs.* the assembled reference sequences, produced here for *Buchnera*, reveals that designed primers did not matched the expected target sequences. Therefore, the lack of amplification is not surprising and should not have been taken as evidence for the lack of these genes in *Buchnera*.

Regarding the experimental analyses, I would argue that in spite of *Wolbachia* being seemingly fixed across different *P. nigronervosa* populations collected in different countries, the fact that this is a facultative endosymbiont cannot be discarded. While it is true that the authors convincingly show that the three populations sampled apparently harbour the same strain of *Wolbachia*, the widespread localisation of this symbiont does not suggest a change of tissue tropism for this strain, but rather what one would expect from a *Wolbachia* bacterium [6–8]. This greatly contrasts the localisation of the nutritional co-obligate *Wolbachia* strain of the bedbug *Cimex lectularius*, where “the infection is substantially restricted to the bacteriome” [9]. Finally, the reported “selective” elimination of the symbionts through antibiotic treatment does not in fact conclusively point towards the obligatory nature of *Wolbachia*. This could only be achieved either through the actual selective elimination of *Wolbachia* and the subsequent measure of biological parameters *vs.* controls (e.g. untreated and/or injected with distilled water), or through proving that the antibiotics solely decrease the quantity of *Wolbachia* and do not affect *Buchnera*. The authors base their conclusions on the treatments with two antibiotics: tetracycline and ampicillin. Regarding the former, the authors incorrectly conclude that since all the injected nymphs died before adulthood and dead nymphs were only positive for *Buchnera* in PCR assays, then the antibiotic treatment “eliminated *Wolbachia*, but only decreased *Buchnera* quantity, without eliminating it completely”. However, as the authors themselves mention, “tetracycline has been reported to have negative effect on *Buchnera* as well as on *Wolbachia*”. Therefore, it is reasonable to conclude that the impact of the decrease in *Buchnera* could also explain the observed mortality. However, without quantitative PCR and/or microscopy analyses, the impact of the antibiotic treatment on *Buchnera* cannot be correctly assessed. In regard to the ampicillin treatment, similar concerns arise. The fact that the authors could only detect *Buchnera* but failed to measure the impact on the abundance of this symbiont in the dead nymphs raises concerns regarding the decrease in *Buchnera* possibly being the responsible for the observed mortality.

## Conclusion

In brief, De Clerck et al. [1] provide no evidence that the apparent fixation of *Wolbachia* in the analysed populations of *P. nigronervosa* is due to this symbiont being a nutritional co-obligate in this aphid. The re-analysis of the sequencing data revealed the genome-based conclusions for a nutritional co-obligate symbiosis in *P. nigronervosa* were erroneous. Additionally, inspection of the primer sequences revealed that most primer pairs do not match the targetted sequences in *Buchnera* from *P. nigronervosa*. Finally, the antibiotic treatment results have alternative explanations, which the authors failed to discuss, and that cannot be discarded without more sensitive analyses. Regardless, *Wolbachia*’s apparently consistent presence could nonetheless be explained by other processes, such as some sort of reproductive manipulation, protection against viral infections, or the aphid strains sampled actually belonging to one, or a few, “superclone(s)” originally infected with closely related *Wolbachia* endosymbionts.

## Materials and methods

### Sequencing data collection and genome assembly

The tree sequencing libraries (SRR1661114, SRR1662246, SRR1662249) from De Clerck et al. [1] were downloaded from the NCBI SRA database (accession SRX766492) from BioProject (https://www.ncbi.nlm.nih.gov/bioproject/268300, last accessed April 15, 2019). Inspection of the downloaded fastq files with fastQC v0.11.7 (https://www.bioinformatics.babraham.ac.uk/projects/fastqc/, last accessed April 15, 2019) revealed contamination of the reads with TruSeq adapter indexes 4, 5, and 6, as well as an unknown T+G-rich adapter (“TGTGTTGGGTGTGTTGGGTGTGTTGGGTGTGTTGGGTGTGTTGGGTGTGT”). To trim these adapters from the read datasets and perform quality trimming, I used fastx_clipper followed by fastx_quality_trimmer which performed right-tail quality trimming (using a minimum quality threshold of 20) and reads shorter than 50 bp were dropped (options -t 20 -l 50). Additionally, PRINSEQ v0.20.4 [10] was used to remove reads containing undefined nucleotides as well as separate those left without a pair after the filtering and trimming process. Assembly of the three libraries was done using SPAdes v3.10.1 [11] with *k*-mers 33, 55, and 77.

### Binning of assembled sequences to *Buchnera* and *Wolbachia*

First, all scaffolds shorter than 200 bp and with an average *k*-mer coverage lower than 3 were dropped. This resulted in 10,097 (3.74% of the total number of scaffolds) which were then used for a BLASTX-based binning. This was done by building a database of the proteomes of the Pea aphid and a set of *Wolbachia* and *Buchnera* bacteria. A separate database of the mitochondrion proteins encoded by the aphid *Schizaphis graminun* was built to detect mitochondria sequences. All these protein datasets were available before the time of publication of De Clerck et al. [1]. Accession numbers and information on strains can be found in supplementary table S4 (Additional bluefile 1). A BLASTX search against the aforementioned databases was done using the filtered scaffolds as query (-soft_masking true -seg yes -max_target_seqs 10000 -evalue 1e-3) followed by assigning the scaffold to the organism with the best BLASTX hit to each scaffold. These bins were then filtered by setting a minimum *k*-mer coverage threshold of 10 for both *Buchnera* and *Wolbachia*. This resulted in 135 and 1309 scaffolds being assigned to each bacterium, respectively.

### Identification of “missing” and “nutritional genes” in *Buchnera*

For verifying the absence of the genes claimed as missing by De Clerck et al. [1] and the “nutritional genes”, I collected the amino acid sequences of these genes from *Buchnera* strain 5A, APS, and Bp. Then, a TBLASTN search of the aforementioned sequences *vs.* both the total filtered scaffolds and the *Buchnera* scaffold bin (-evalue 1e-03 -db_gencode 11) was performed. Afterwards, manual verification of each hit was performed using UGENE v1.29.0 and then BLASTX on the on-line BLAST server from NCBI *vs.* the nr database. If the gene was not found in the *Buchnera* scaffold bin, then the gene was verified from the TBLASTN *vs.* the filtered scaffolds. This revealed the misassignment of “NODE_9” to the *Wolbachia* bin. This manual curation also revealed remnants of the “TGTGTTGGGTGTGTTGGGTGTGTTGGGTGTGTTGGGTGTGTTGGGTGTGT” sequence inside the *lipA* and *pgi* genes. Further manual inspection of the reads revealed a consistent insertion of this sequence in a specific place of each gene, revealing the biassed insertion of the contaminant. Additionally, an alignment of each read library *vs.* the abovementioned *Buchnera* genes from *P. nigronervosa* was done with Bowtie2 v2.3.4.1 [12] for visualisation purposes. To avoid reads from *Wolbachia* mapping to sequences of orthologous genes from *Buchnera*, the *Wolbachia* scaffolds were also included in the Bowtie2 mapping.

The suitability of the reported primers in De Clerck et al. [1] (found in “Additional file 1: Table S1”) was judged by aligning them *vs.* the gene sequence identified and reported in the present work using AliView v1.26 [13].

## Supplementary information

**Additional file 1** Supplementary tables S1-S4. XLS-formatted supplementary tables S1-S4, including descriptions.

## Abbreviations

EAAs: Essential amino acids

## References

[1] De Clerck C, Fujiwara A, Joncour P, Léonard S, Félix M-L, Francis F, Jijakli MH, Tsuchida T, Massart S (2015). A metagenomic approach from aphid’s hemolymph sheds light on the potential roles of co-existing endosymbionts. Microbiome.

[2] Lai CY, Baumann P, Moran NA (1996). The endosymbiont (*Buchnera* sp.) of the aphid *Diuraphis noxia* contains plasmids consisting of *trpEG* and tandem repeats of *trpEG* pseudogenes. Appl Environ Microbiol.

[3] Baumann Linda, Clark Marta A., Rouhbakhsh Dadbeh, Baumann Paul, Moran Nancy A., Voegtlin David J. (1997). Endosymbionts ( Buchnera ) of the Aphid Uroleucon sonchi Contain Plasmids with trpEG and Remnants of trpE Pseudogenes. Current Microbiology.

[4] Meyer F, Paarmann D, D’Souza M, Olson R, Glass E, Kubal M, Paczian T, Rodriguez A, Stevens R, Wilke A, Wilkening J, Edwards R. The metagenomics RAST server – a public resource for the automatic phylogenetic and functional analysis of metagenomes. BMC Bioinformatics; 1:386. 10.1186/1471-2105-9-386. 10.1186/1471-2105-9-386 PMC2563014 18803844

[5] Aziz Ramy K, Bartels Daniela, Best Aaron A, DeJongh Matthew, Disz Terrence, Edwards Robert A, Formsma Kevin, Gerdes Svetlana, Glass Elizabeth M, Kubal Michael, Meyer Folker, Olsen Gary J, Olson Robert, Osterman Andrei L, Overbeek Ross A, McNeil Leslie K, Paarmann Daniel, Paczian Tobias, Parrello Bruce, Pusch Gordon D, Reich Claudia, Stevens Rick, Vassieva Olga, Vonstein Veronika, Wilke Andreas, Zagnitko Olga (2008). The RAST Server: Rapid Annotations using Subsystems Technology. BMC Genomics.

[6] Gómez-Valero Laura, Soriano-Navarro Mario, Pérez-Brocal Vicente, Heddi Abdelaziz, Moya Andrés, García-Verdugo José Manuel, Latorre Amparo (2004). Coexistence of Wolbachia with Buchnera aphidicola and a Secondary Symbiont in the Aphid Cinara cedri. Journal of Bacteriology.

[7] Ahmed Muhammad Z., Li Shao-Jian, Xue Xia, Yin Xiang-Jie, Ren Shun-Xiang, Jiggins Francis M., Greeff Jaco M., Qiu Bao-Li (2015). The Intracellular Bacterium Wolbachia Uses Parasitoid Wasps as Phoretic Vectors for Efficient Horizontal Transmission. PLOS Pathogens.

[8] Schneider DI, Parker AG, Abd-alla AM, Miller WJ. High-sensitivity detection of cryptic *Wolbachia* in the African tsetse fly (*Glossina* spp.)BMC Microbiol; S1:140. 10.1186/s12866-018-1291-8. 10.1186/s12866-018-1291-8 PMC6251158 30470185

[9] Hosokawa T., Koga R., Kikuchi Y., Meng X.-Y., Fukatsu T. (2009). Wolbachia as a bacteriocyte-associated nutritional mutualist. Proceedings of the National Academy of Sciences.

[10] Schmieder R., Edwards R. (2011). Quality control and preprocessing of metagenomic datasets. Bioinformatics.

[11] Bankevich Anton, Nurk Sergey, Antipov Dmitry, Gurevich Alexey A., Dvorkin Mikhail, Kulikov Alexander S., Lesin Valery M., Nikolenko Sergey I., Pham Son, Prjibelski Andrey D., Pyshkin Alexey V., Sirotkin Alexander V., Vyahhi Nikolay, Tesler Glenn, Alekseyev Max A., Pevzner Pavel A. (2012). SPAdes: A New Genome Assembly Algorithm and Its Applications to Single-Cell Sequencing. Journal of Computational Biology.

[12] Langmead Ben, Salzberg Steven L (2012). Fast gapped-read alignment with Bowtie 2. Nature Methods.

[13] Larsson Anders (2014). AliView: a fast and lightweight alignment viewer and editor for large datasets. Bioinformatics.

